# Evaluating antibiotic use and developing a tool to optimize prescribing in a family-centered HIV clinic in Eswatini

**DOI:** 10.1371/journal.pone.0244247

**Published:** 2021-01-07

**Authors:** Tara E. Ness, Ashish E. Streatfield, Tandzile Simelane, Abiy Korsa, Sandile Dlamini, Danielle Guffey, Bhekumusa Lukhele, Alexander W. Kay

**Affiliations:** 1 Baylor College of Medicine, Houston, Texas, United States of America; 2 Department of Pediatrics, Texas Children’s Hospital, Houston, Texas, United States of America; 3 Community HIV/AIDS Mitigation Project, United States Peace Corps, Mbabane, Eswatini; 4 Baylor Center of Excellence, Mbabane, Eswatini; 5 Global Tuberculosis Program, Texas Children’s Hospital, Houston, TX, United States of America; Manipal College of Medical Sciences, NEPAL

## Abstract

In a human immunodeficiency virus (HIV) clinic for children and their families in Eswatini, we sought to understand the use of antibiotics and identify specific areas for improvement. We performed a retrospective patient chart review as part of a quality improvement (QI) initiative to assess antimicrobial use before and after implementation of a standardized antimicrobial guide. For each prescribing period, 100 random patient encounters were selected for review if the indication for antibiotics, duration, and dose were consistent with World Health Organization (WHO) guidelines. Two physicians reviewed each encounter using a structured abstraction tool, with a third resolving discrepancies. Results were analyzed using a chi-square test of proportions and a structured survey was performed to assess perceptions of the guide. After the implementation of an antimicrobial guide, there was a significant decrease in the proportion of clinic visits with an antibiotic prescribed (p < 0.001). Incorrect indication for antimicrobial use decreased from 20.4% in the initial period to 10.31% and 10.2% but did not reach significance (p = .0621) in the subsequent periods after implementation. Incorrect dose/duration decreased from 10.47% in the initial period to 7.37% and 3.1% in the subsequent periods, but this was also was not significant (p = 0.139). All prescribers who completed the survey felt that it positively impacted their prescribing. Our study found that an antimicrobial guide reduced and improved the prescription of antimicrobials, demonstrating practical solutions can have a lasting impact on prescribing in low resource settings.

## Introduction

Global antimicrobial consumption is estimated to have increased by 36% from 2000 to 2010 [1] and by 65% from 2010 to 2015, driven primarily by low- and middle- income countries (LMICs) [2]. Of patients who were prescribed at least one antimicrobial, only 19.8% received a targeted antibacterial treatment [3] and in both high-income countries (HICs) and LMICs, use of “last resort” antibiotics has increased [2]. Overuse and misuse of antibiotics is linked to increasing antibiotic resistance [4, 5] which, in turn, leads to higher mortality and increased medical costs [6–8]. This is especially problematic in sub-Saharan Africa, where people with HIV, including children, are particularly susceptible to drug-resistant infections [9–12]. In addition, an incorrect antibiotic choice can delay a patient’s recovery and cause unnecessary side effects. Despite these challenges, antibiotics are also an important tool that have led to significant decreases in mortality since their discovery.

Antibiotic prescribing, especially in LMICs, can be challenging. This is due to differing levels of prescriber training, limited access to resources for correct antibiotic choice and dosing, and stock outs or general unavailability of optimal antibiotic choices. In order to evaluate antibiotic prescribing patterns on a facility level, the World Health Organization (WHO) has developed prescribing indicators for health care facilities (WHO-drug use indicators). These indicators, aimed at increasing attention on antibiotic stewardship at a local level, have resulted in a number of hospital-based interventions across sub-Saharan Africa with an observed decrease in antibiotic use without increasing morbidity and mortality at those institutions [13, 14]. While these studies show the effectiveness of antibiotic stewardship programs at secondary and tertiary care facilities, there is very little literature regarding similar programs at primary care facilities, where the majority of provider-patient interactions and antibiotic prescriptions take place in sub-Saharan Africa [15, 16].

Many LMICs in sub-Saharan Africa lack the ability for targeted diagnostic testing, such as blood cultures, rapid antigen tests, or nucleic acid amplification tests, in order to target antibiotic prescribing [13, 17, 18]. As a result patients are treated based on infectious syndromes. Widespread practice of syndromic medicine can also result in over-prescription of antibiotics in an effort to safeguard patients from possible infections [16, 19].

Our quality improvement project sought to investigate the prescribing patterns at a family-centered HIV clinic in Eswatini and investigate whether a targeted “Antimicrobial Guide” providing a quick reference for antimicrobial choices, weight-based dosing of antimicrobials, and the location-specific drug formulations available, could improve use of antibiotics. To our knowledge, this is the first quality improvement (QI) project on antimicrobial stewardship in an outpatient clinic in Eswatini, though the approach could be used in other geographic and treatment settings.

## Materials and methods

### Project setting and population

This study took place at the Baylor College of Medicine-Bristol-Myers Squibb Children's Clinical Centre of Excellence (COE) in Mbabane, Eswatini which serves children with HIV and their families. Eswatini has the highest prevalence of HIV in the world among adults aged 15 to 49 years at 27.3% with 13,000 children (age 0–14) living with HIV [20]. Although located within a city, the outpatient clinic provides services to rural inhabitants and is a referral center for many rural clinics. Baylor-Eswatini is the largest pediatric antiretroviral (ARV) provider for children in the country, treating approximately 40% of children with HIV in Eswatini through its various clinics. Pharmaceuticals, including antibiotics, are dispensed on-site and are free of charge. The Baylor COE clinic in Mbabane currently has 3066 active patients and averages 22500 patient visits per year over the last ten years. Of the 3066 patients, 2954 are HIV positive.

### Study design

This was a pre-post quasi experimental study to assess the efficacy of an antimicrobial prescribing guide and standardized training. A retrospective patient chart review of encounters was conducted in three distinct time periods, from January 1 to April 30, 2019 (Period 1), from July 1 to September 30, 2019 (Period 2), and from October 1 to January 17, 2020 (Period 3). The antimicrobial guide was introduced in May 2019 and continues to be used. Two time periods following the guide introduction were chosen to determine if changes in prescribing were sustained. The percentage of encounters with antibiotic prescribing in each period was calculated from the electronic medical records. Trimethoprim/sulfamethoxazole (TMP-SMX) was excluded as it is usually prescribed for prophylaxis in HIV patients. The overall percent of encounters in each time period in which antibiotics were prescribed was calculated from this data. From those encounters in which antibiotics were prescribed (5471 in Period 1, 6293 in Period 2, and 6355 in Period 3), 100 encounters were randomly selected, via a random number generator, to review the indication for antibiotics (e.g. pneumonia, skin infection), and the prescribed dose and duration. Dose and duration were interpreted as a single parameter due to their being acceptable dose/duration alternatives for an antibiotic for a syndrome (e.g. 2 grams of metronidazole once versus 500 mg twice daily for 7 days for *Trichomonas vaginalis* infection). Clinician prescribing was evaluated against the guide as well as current medical organization guidelines such as the Eswatini Ministry of Health, the WHO and the American Academy of Pediatrics. Incorrect indication was any prescription in which the prescribed antibiotic did not treat the most likely organisms for that syndrome or was unnecessary (for example, prescribing any antibiotic for a viral gastroenteritis). Each encounter was reviewed by at least two clinicians (TN, TS, and/or AK2) working independently. Any discrepancies or uncertainties were resolved after review and discussion with the third reviewer and group consensus.

### Antimicrobial guide design

**Fig 1:**
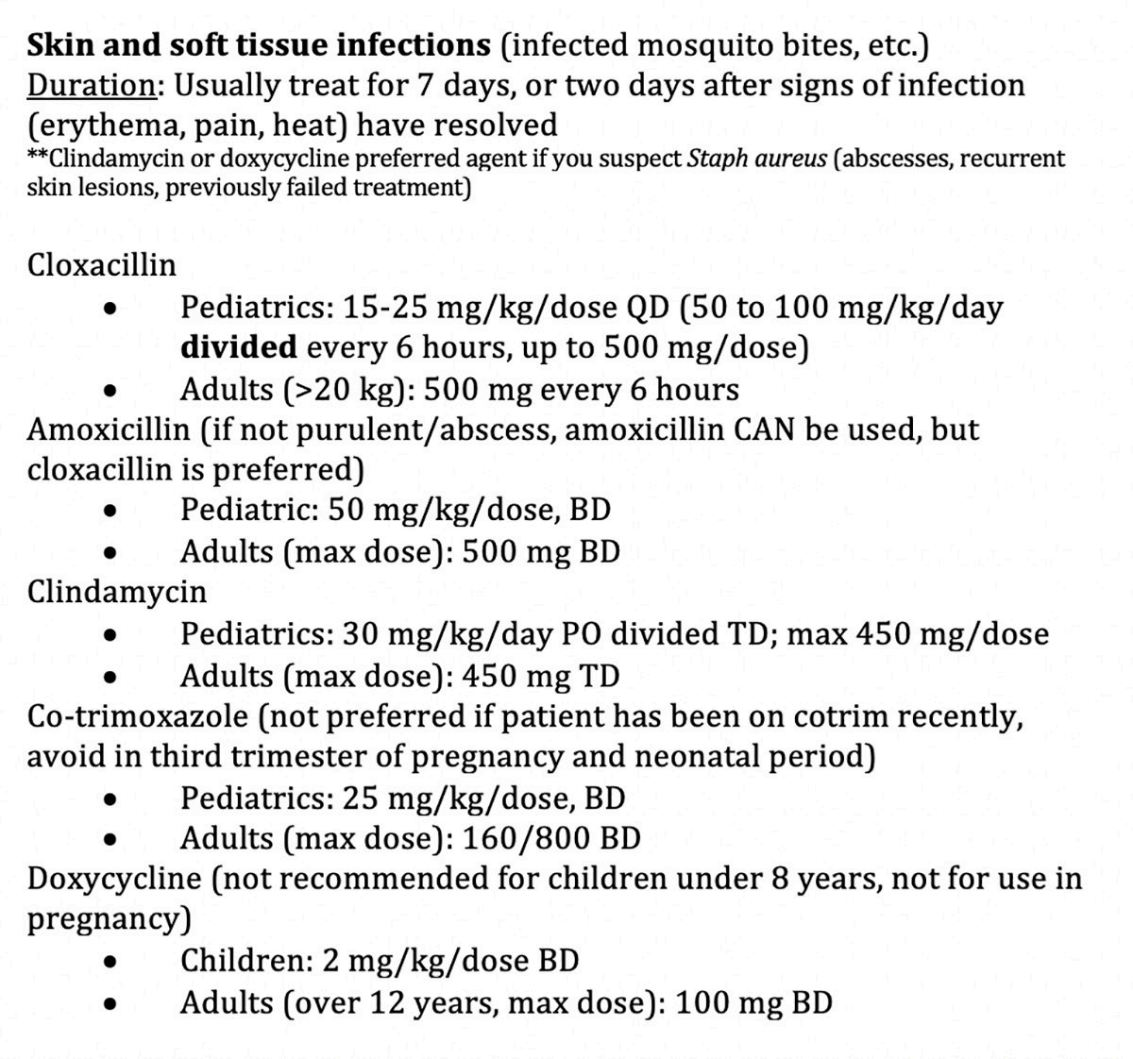
Excerpt from antimicrobial guide showing specific antibiotic guidance by clinical symptoms.

**Fig 2:**
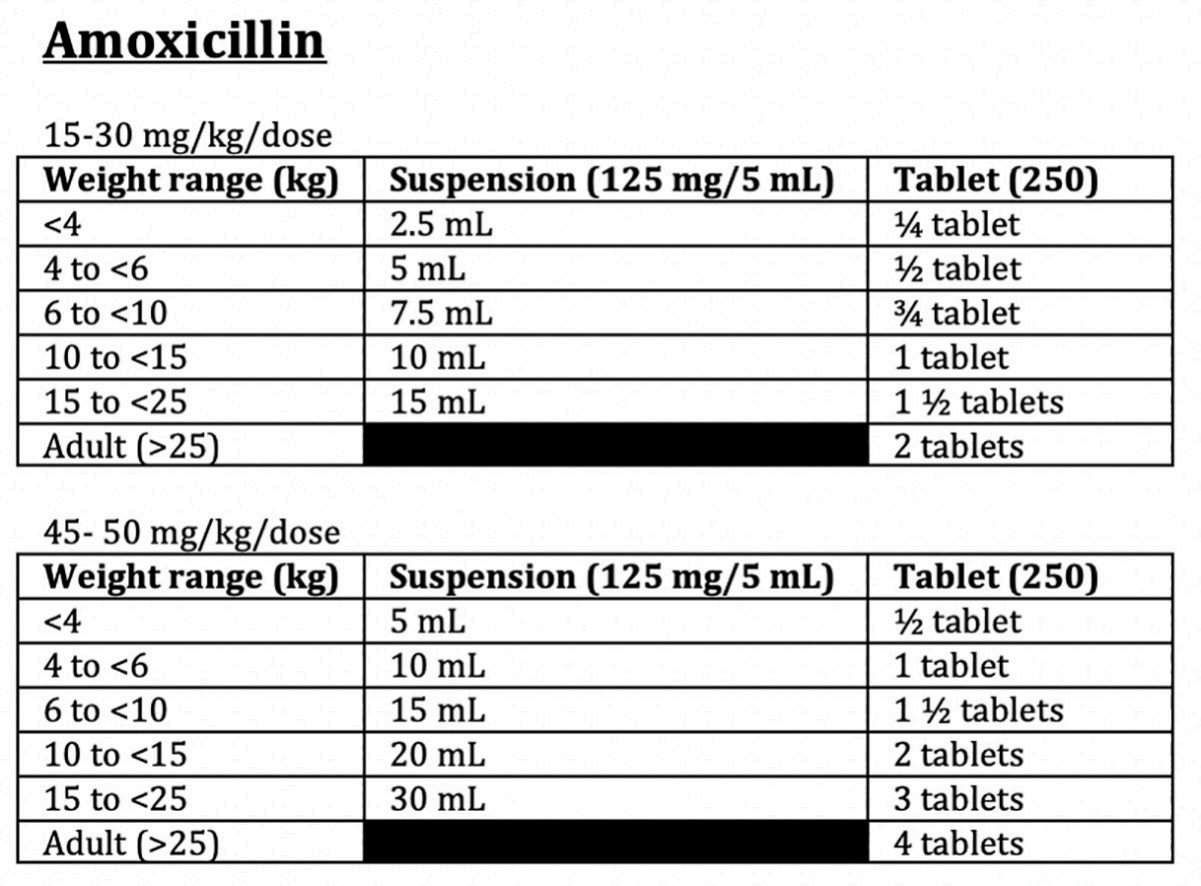
Excerpt from antimicrobial guide showing weight-based dosing charts for ease of prescriber use.

Creation of the Antimicrobial Guide was a cooperative effort between clinical staff and the pharmaceutical department. After the first round of patient encounters was reviewed (January 1 to April 30, 2019), TN (clinical staff), AK1 (pharmaceutical staff), and AK2 (clinical staff) collaborated to create an Antimicrobial Guide for the clinic, based on country guidelines, WHO guidelines and other available resources. The guide was not meant to be all encompassing but tailored to provide guidance on commonly encountered diseases or clinical syndromes (skin and soft tissue infections, respiratory infections, urogenital infections, etc.) and more rare diseases requiring specific therapy. The guide included the type and dosage of antibiotic recommended for adults and children and reflected the medications available in the clinic formulary. Alternative recommendations were offered in order to account common medication shortages. Additionally, weight-based charts for specific doses of antibiotics were provided for quick reference to increase efficiency of providers. Figs 1 and 2 provide examples of guidance included on the Antimicrobial Guide.

### Staff training

Standardized training on the use of the antimicrobial guide was provided for clinical staff with implementation of the guide in May 2019 and on an as-needed basis through targeted “patient-specific” teaching in response to the interim reviews. The Antimicrobial Guide was placed in each clinic consultation room as well as the Pharmacy for ease of reference and continued staff to staff education. Staff were provided the opportunity for feedback on the guide which was integrated into the guide on an ongoing basis.

### Control for bias

To control for provider bias in interpretation of “correct” versus “incorrect” prescribing choices by time period, patient encounters reviewed by the initial two physicians were randomly mixed and provided to a third physician reviewer, blinded to the time point of the prescription. This third provider agreed with the interpretation of prescribing choices indicating the clinicians were not influenced by the desire to show improvement in prescribing patterns after the implementation of the antibiotic guide. Although it was impossible to blind the first two physicians to the name of the individual who prescribed the antibiotic reviewed based on the set-up of our electronic medical record, the third physician was always blinded to this and no physician reviewed their own prescribing.

### Questionnaires

In addition to objective measures of improved prescribing, a qualitative assessment of how providers felt about the antimicrobial guide was conducted through an anonymous semi-structured survey. The questionnaire was provided to ten clinicians (doctors or nurses) and two pharmaceutical staff with a 100% response rate. The questionnaire asked whether the provider used the antibiotic guide, how often the antibiotic guide was used, how it impacted care, what types of diseases and antibiotics the guide was used most often for, and if they would recommend the guide to other clinics. The questionnaire is included under S1 File.

### Data analysis

Results of the retrospective chart reviews were analyzed to determine the impact of the antimicrobial guide by comparing the results (% of patient encounters in which an antibiotic was prescribed, % of encounters in which an antibiotic was prescribed without a clear indication, % with an incorrect indication for a particular antibiotic(s), and % of encounters with an incorrect dose and/or duration) of the initial time period prior to guide implementation to the subsequent time periods after the intervention. Chi-square tests were conducted to analyze the impact of the intervention by comparing the results of three time periods; the initial time period prior to guide implementation and two subsequent time periods after guide implementation. If the chi-square tests were significant for all three periods, the pairwise chi-square tests were conducted to compare the first and second period, the first and third period, and then the second and third period. The p-values from pairwise chi-square tests were adjusted by the Holm method to counteract the problem of multiple comparisons. A p-value less than .05 was considered statistically significant. Two time periods post implementation were selected in order to see if the impact was sustained. Fisher’s exact test was used, instead of chi-square test, when small cell sizes (smaller than 5) were present. The results of the provider survey were summarized but no statistical analysis was performed.

### Ethical considerations

This study utilized de-identified patient data extracted from the electronic medical record (EMR) database of the Baylor COE with the assistance of the data manager with permission to collect the data from all relevant parties, including the clinic director and pharmacotherapy committee. It was considered a quality improvement measure with no impact to human subjects so did not require informed consent or specific Institutional Review Board approval.

## Results

### Patient demographics

Patient demographics from the three prescribing periods are summarized in Table 1. Overall, there was no significant difference in the age or gender demographics between the three periods. The mean age, median age with interquartile range (IQR), complete age range, and proportion of females for each period for all encounters and antibiotic encounters are provided. There was no significant difference in age or gender distribution in the prescribing periods or when comparing all encounters to encounters where antibiotics were prescribed.

**Table 1:** Patient demographics by prescribing period.

Patient Characteristic	Period 1	Period 2	Period 3
Mean age, all encounters	22 years	24 years	24 years
Mean age, antibiotic encounters	26 years	28 years	29 years
Median age (IQR), all encounters	19 years (10–35)	20 years (11–36)	20 years (12–36)
Median age (IQR), antibiotic encounters	21 years (15–38)	30 years (18–39)	31 years (18–39)
Proportion female, all encounters	59.70%	61.33%	61.29%
Proportion female, antibiotic encounters	66.54%	71.20%	69.96%

### Impact of antibiotic guide

In all three prescribing periods, metronidazole, erythromycin, and doxycycline were the most commonly prescribed antibiotics. The most common diseases/clinical syndromes included skin and soft tissue infections, respiratory infections, and urogenital infections. There did not appear to be any increase or decrease in any specific infections/clinical syndromes or antibiotics. In the initial prescribing period (prior to implementation of the Antimicrobial Guide and teaching), 9.83% (538/5471) of patient encounters involved the prescription of an antibiotic. In subsequent time periods (post-implementation) 6.99% (440/6293) and 7.02% (446/6355) of encounters involved an antibiotic prescription. Using a chi-square test comparing all three time periods gave a p-value (p < 0.001), meaning at least one of the periods was significantly different from other periods. The p-value for Period 1 and Period 2 was <0.001, Period 1 and Period 3 was <0.001, and Period 2 and Period 3 was 0.9817.

Table 2 summarizes the findings by prescribing period. There was no significant difference among all three periods (p = 0.253) for prescriptions without an indication documented either in the prescription text or provier note. There was no significant difference among all three periods for incorrect antibiotic indication (p = 0.062) or incorrect dose or duration (p = 0.1394).

**Table 2:** Summary of assessments by period. Please note that some assessments are not out of 100 due to the subtraction of “no indication” or “not applicable” from that particular column.

Assessment	Period 1	Period 2	Period 3	p-value*
Encounters without indication	7% (7/100)	3% (3/100)	2% (2/100)	0.253
Incorrect antibiotic indication	20.43% (19/93)	10.31% (10/97)	10.20% (10/98)	0.062
Incorrect antibiotic dose or duration	10.47% (9/86)	7.37% (7/95)	3.10% (3/97)	0.1394

*Fisher’s exact test.

### Provider questionnaire

Ten prescribers and two pharmacists voluntarily and anonymously completed the Provider Questionnaire. All reported using the guide at least weekly. The prescribers reported using the antibiotic guide most for clinical symptoms involving skin/soft tissue infections (the highest at 11/12 survey respondents), ear/nose/throat infections (8/12), pneumonia (6/12) and sexually transmitted infections (6/12). When asked about specific antibiotics the guide was referenced for, cloxacillin was the highest use (8/12 survey respondents), followed by amoxicillin (7/12), metronidazole (5/12) and acyclovir (5/12). One provider commented “It makes my job easier; I spend less time now thinking about choice of antibiotic to use.” Another stated “It’s really helpful and straight to the point.” Of the two pharmacists, one felt that the guide had led to 50% decrease in number of daily phone calls to ask prescribers to correct an antibiotic prescription and the other felt there was a 75% decrease in daily call volume.

## Discussion

Antibiotics were a ground-breaking advancement in the fight against infectious diseases and are a vital tool in the armamentarium of clinicians. Despite this benefit, overuse and misuse of antibiotics threaten to undermine their effectiveness due to the development of drug-resistant bacteria [21]. The importance of antibiotic stewardship has been highlighted by organizations such as the WHO, as evidenced by the production of toolkits and a coordinated response to antimicrobial resistance through various partnerships [22]. In addition to global efforts, interventions aimed at the facility level can help improve antibiotic use and lead to better patient outcomes in low-resource settings.

The development of the guide was informed by antimicrobials that were available at the clinic, so prescribers would not have to consider antibiotic choices that were unavailable. We believe pragmatic contextual adaptation of more universal tools for stewardship can contribute to efficacy. In addition, when possible we offered several options for each clinical syndrome so that in the case of a pharmaceutical stock out, there would be other options available. Lastly, we recognized that weight-based dosing can be complicated and time consuming, so we provided easy-to-use dosing charts for the formulations available within our pharmacy.

We found a statistically significant decrease in patient encounters in which an antibiotic was prescribed, meaning that providers were more thoughtful about whether an antibiotic was necessary. This continued for both time periods following guide implementation indicating that this change was sustained. We observed some evidence of a decrease in the number of antibiotics prescribed without a documented indication, and a reduction in incorrect antibiotic choices and dosing; however, our ability to detect statistically meaningful changes was limited by the sample size evaluated. Engagement of pharmacists in the intervention also may have limited our ability to detect ongoing improvements as pharmacists contacted prescribers to provide stewardship throughout the intervention.

Previous interventions on behalf of antimicrobial stewardship have primarily been based at the provider level, but pharmaceutical and patient level interventions have also been shown to be beneficial. One meta-analysis of educational interventions to improve antibiotic prescription and dispensing showed that of 47 studies conducted in the outpatient setting, 33 utilized the dissemination of printed/audiovisual educational materials similar to the antimicrobial prescribing guide used in this study [23]. Another review, specifically looking at inpatient antibiotic stewardship interventions in hospitals in low-and middle-income countries, showed the creation of treatment guidelines decreased daily doses of antibiotics per 100 bed-days per month, which was demonstrated in studies performed in India and Indonesia [24]. These results are consistent with our own findings in the outpatient setting that dissemination of a guide and structured teaching led to decreased prescribing of antibiotics. The guide itself provided some guidance under particular conditions, such as diarrhea, encouraging the prescriber to consider that supportive treatment alone may be sufficient if the clinical symptoms were consistent with a viral process. We feel that this is the most likely reason for the dramatically decreased prescribing of antibiotics, though overall improvement in prescriber comfort with antibiotics and recognition of viral versus bacterial syndromes may also play a role. Another significant finding was the reduction in daily call volume reported by the pharmaceutical staff to correct prescribing errors providers made. These calls disrupt both the efficiency of the pharmacy as well as the provider as they must go back to correct their mistakes when they may be in the middle of a patient encounter. Reducing this allows the providers to have uninterrupted visits with subsequent patients and the pharmaceutical staff to focus on dispensing medicines rather than trying to get ahold of providers.

Our study has several limitations. It is possible that improvements in prescribing patterns were seen with our chart review due to prescribers being aware that their antibiotic use would be reviewed. While no formal announcement was made to prescribers that chart reviews were being done, it was also not kept secret and it is possible that prescribers heard about the study and adjusted their practices. Even if this was the case, provider questionnaires showed that they overwhelmingly utilized the guide and found it helpful, making it more likely that use of the guide resulted in improved prescribing. As mentioned, the guide was introduced in May 2019, and our subsequent reviews did not begin until July 2019 to allow time for prescribers to settle into routines and the subsequent reviews covered a six month period, making it unlikely prescribers maintained a change in behavior for this extended period of time simply based on a fear of being audited (Hawthorne effect). A limitation, as with all printed materials, is that the guide is not easily updated to reflect changes in the clinic formulary or new emerging research. Despite this, we feel that reviewing the guide every 6 months will allow for a functioning, helpful guide without too much of an administrative burden. Data costs and slow internet speeds in many LMICs mean that hard copies of guides are preferred to online tools. In addition to continuing to review the guide on a regular basis, next steps should include reassessing the guide’s impact over the coming years and evaluating the implementation of a similar guide in other settings, such as other outpatient clinics or inpatient settings.

Overall, our study highlights that an outpatient facility-level intervention, in the form of an antimicrobial guide, pharmacist engagement and targeted clinician training on antimicrobial stewardship and infectious syndromes, can have a positive impact on antibiotic prescribing patterns and is deemed a valuable tool by prescribers and pharmacists. Antimicrobial guides and stewardship training should be more broadly implemented in LMICs. Strategies that involve patient engagement should also be considered to improve antibiotic use at the outpatient facility level in LMICs.

## Supporting information

S1 File (PDF)
