# Supplementary material for: Evaluating antibiotic use and developing a tool to optimize prescribing in a family-centered HIV clinic in Eswatini
Source: PLoS One. 2021 Jan 7;16(1):e0244247. doi: 10.1371/journal.pone.0244247 (PMC7790297; doi:10.1371/journal.pone.0244247)
Supplement: S1 File — (PDF) [file pone.0244247.s001.pdf]

Provider Type (doctor/nurse/pharmacist):
Number of years in clinical practice (seeing patients out of school):

1. Do you use the Antibiotic Guide?

Yes No

2a. If yes, how often do you use the guide? (please circle)

Once a week 1-3 times/week 3-5 times/week Once/day Several times a day

2b. If not, why not? (please circle, you may circle more than one)

1. Do not prescribe antibiotics often/check prescriptions often
2. Did not find it useful/applicable
3. Did not need it (already know antibiotic dosages well)
4. Did not know the guide existed
5. I have another reference I use instead
6. Other________________________________

3. What is useful/not useful about the antibiotic guide?

4. What would you change about the guide?

5. Which types of diseases did you use the guide most often for? (please circle, you may circle more than one)

Skin/soft tissue infection Animal bites Ear/nose/throat infections Urinary tract infections

Pneumonia Diarrhea Genital ulcers Herpes zoster Sexually transmitted infections

Other___________________________

6. What types of antibiotics/antivirals did you use the guide the most for? (please circle, you may circle more than one)

Cloxacillin Doxycycline Amoxicillin Ciprofloxacillin Cotrimoxazole Erythromycin

Metronidazole Acyclovir Azithromycin Penicillin Other_____________________

7. Are there any antibiotic/antivirals or diseases that are not on the guide that you wish were?

8. Would you recommend the guide to other clinics?

Yes No

9. Do you feel the guide has made you better are prescribing antibiotics/antivirals?
(For pharmacists, do you feel that you have made less phone calls to correct antibiotic prescribing?)

Yes No

If yes, how has the guide impacted you? (please circle, you may circle more than one)

More accurate dosing More accurate antibiotic choice Using antibiotics less (more stewardship)

Other______________________________

10. Any other comments?

11. For pharmacists, what has the impact been on the number of phone calls you have to make to providers to correct antibiotic choice or antibiotic dosing since the guide was released? (Providers skip this question)

I make more calls now I call about the same amount Slight reduction (25%) in calls

50% decrease in calls Huge decrease (75%) in calls

Other_______________________________
